# Introducing a drift and diffusion framework for childhood growth research

**DOI:** 10.12688/gatesopenres.13123.2

**Published:** 2020-11-26

**Authors:** Fraser I Lewis, Godfrey Guga, Paschal Mdoe, Esto Mduma, Cloupas Mahopo, Pascal Bessong, Stephanie A Richard, Benjamin J J McCormick

**Affiliations:** 1 Independent Researcher, Utrecht, The Netherlands; 2 Haydom Lutheran Hospital, Haydom, Tanzania; 3 University of Venda, Thohoyandou, 0950, South Africa; 4 Fogarty International Center, Bethesda, MD, USA

**Keywords:** child growth, stochastic differential equations, dynamic modelling, MAL-ED

## Abstract

**Background**: Growth trajectories are highly variable between children, making epidemiological analyses challenging both to the identification of malnutrition interventions at the population level and also risk assessment at individual level. We introduce stochastic differential equation (SDE) models into child growth research. SDEs describe flexible dynamic processes comprising: drift - gradual smooth changes – such as physiology or gut microbiome, and diffusion - sudden perturbations, such as illness or infection.

**Methods**: We present a case study applying SDE models to child growth trajectory data from the Haydom, Tanzania and Venda, South Africa sites within the MAL-ED cohort. These data comprise n=460 children aged 0-24 months. A comparison with classical curve fitting (linear mixed models) is also presented.

**Results**: The SDE models offered a wide range of new flexible shapes and parameterizations compared to classical additive models, with performance as good or better than standard approaches. The predictions from the SDE models suggest distinct longitudinal clusters that form distinct ‘streams’ hidden by the large between-child variability.

**Conclusions**: Using SDE models to predict future growth trajectories revealed new insights in the observed data, where trajectories appear to cluster together in bands, which may have a future risk assessment application. SDEs offer an attractive approach for child growth modelling and potentially offer new insights.

## Introduction

Assessing and analysing growth is a key activity in paediatric epidemiology, building on centuries of research ^1^. Anthropometrics are easy to measure with basic equipment and the results are both immediate and meaningful with standardised reference measurements representative of unconstrained growth available from the World Health Organization (WHO) ^2^. This makes observations of weight, length, and weight-for-length attractive as measures of a child’s long- and short-term health ^3, 4^. One of the main challenges to analysing child growth data is that individual growth trajectories display highly variable and complicated dynamic behaviour, differing markedly between children, even from the same geographic and socio-economic group. As such, developing growth models from which actionable insights can be extracted – such as identification of interventions at the population level or predictive risk assessments at individual child level – is both methodologically and practically challenging. Here we introduce a new methodology, stochastic differential equation (SDE) ^5^ models, into child growth research.

SDEs describe highly flexible dynamic processes comprising of two components: drift – gradual smooth changes, which could reflect developmental biological aspects such as physiology, nutrition or gut microbiome ^6^; and diffusion – sudden short-term perturbations or shocks – like seasonal food insecurity, illness ^7^ or infection ^8^. This stochastic behaviour could potentially help explain the large variability seen in growth trajectories. SDEs are extensively used in certain specialised applications, most notably in financial modelling ^9, 10^, to cope with the complicated dynamics of stock price movements. Some case studies utilizing SDEs exist in medicine and biology ^11^, but they are not yet a part of a typical epidemiologist’s or statistician’s modelling toolbox.

To date, a wide range of different statistical curve-fitting methodologies have been applied to child growth trajectories, from common classical approaches such as hierarchical linear mixed models ^12^, through to methods such as linear spline multilevel/broken stick ^13^ models, SITAR ^14^ growth curves, dynamic regression models ^15^ and functional principle component models ^16^. SDEs are not curve fitting models but continuous time stochastic processes capable of rich dynamic behaviour. Given this, SDEs can enhance mechanistic interpretation of the drivers of variability (both long- and short-term) in growth in addition to improving forecasts of growth by more accurately capturing external sources of variability and uncertainty.

In the Methods section we provide a brief overview of SDE models. We present a minimum of theory, using instead two empirical case studies to introduce the key features of SDE modelling and how it can be readily applied in practice. In the Results section we present a more complex case study, including quantifying the impact of covariates on growth, using data from the two African sites of the MAL-ED study ^17^. We conclude with a brief discussion of the opportunities for the application of SDEs in child growth research and outline some existing challenges. The computer code required to repeat the modelling results presented are provided as *Extended data* ^18^.

## Methods

### Data sets and initial exploration

We use individual child data from the MAL-ED study, whose protocols, methodology and aggregate growth results have been presented previously ^8, 17^. MAL-ED was initiated as a multi-country cohort study located across eight low- and middle-income sites with historically high incidence of diarrhoeal disease and undernutrition, with a research focus on investigating determinants of development in children from birth through early years. Ethical approval was obtained from the National Institute of Medical Research for Tanzania (NIMR/HQ/R.8a/Vol.IX/858 and NIMR/HQ/R.8c/Vol.II/1034) and University of Venda Ethics Committee for South Afirca (SMNS/09/MBY/004). Approval was additionally given by the institutional review board of the University of Virginia, USA and all methods used in this study followed the relevant guidelines and regulations. Informed consent was taken from parents of all children prior to enrolment.

**Figure 1.**
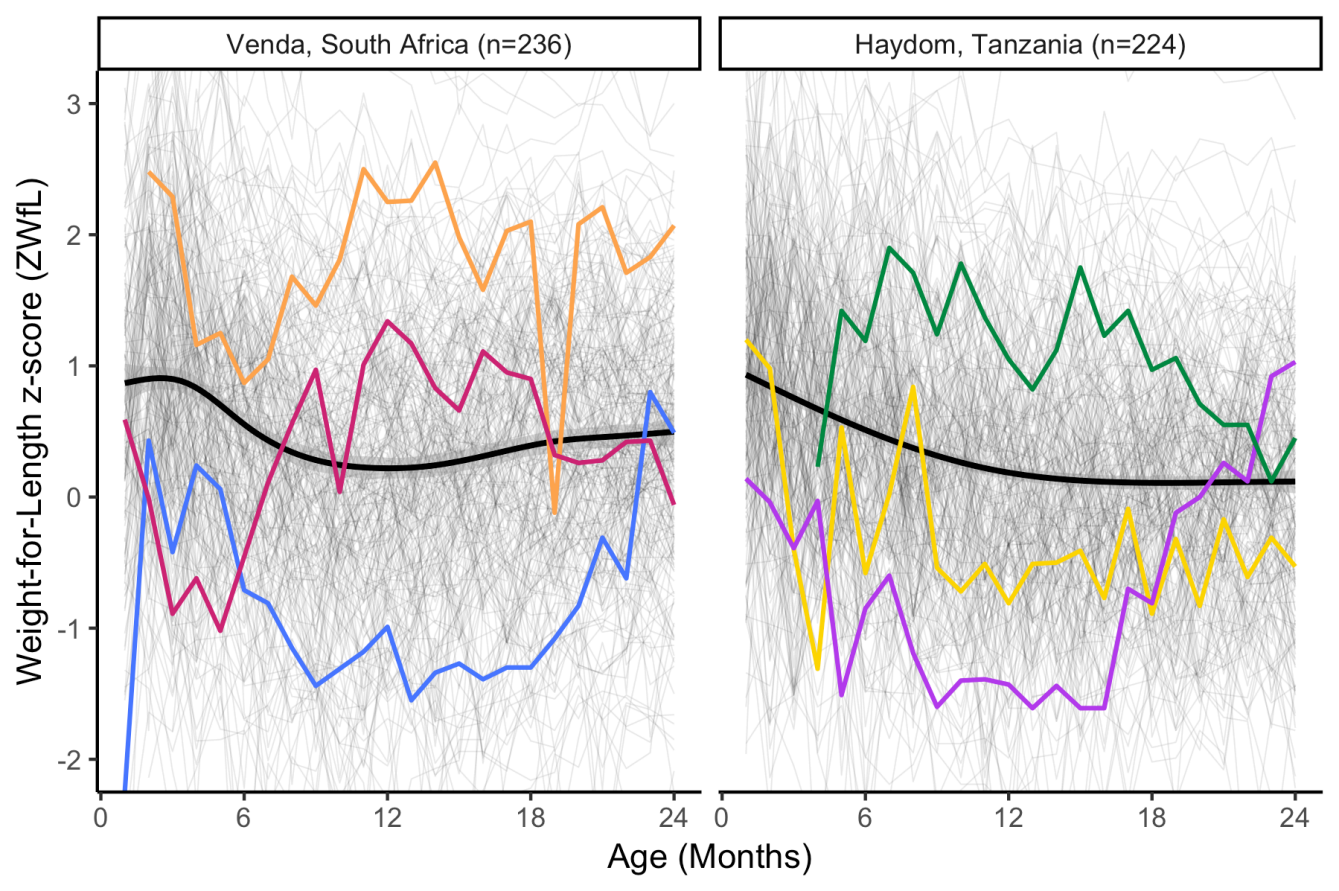
Observed weight-for-length trajectories for children from Venda and Haydom, 0–24 months, each thin grey line is an individual child and the thick black line is the population mean. Large within and between child variability is clear, with three random children highlighted in each site.

In our case studies we use data from Haydom, Tanzania ^19^ (n = 224) and Venda, South Africa ^20^ (n = 236). Our focus here is on anthropometric data from ages 0–24 months where each child included in these analyses had between 20 and 25 monthly observations (within a window of ±14 days), with 83% of children in Haydom and 86% in Venda having at least 24 observations. Weight and length, collected by trained fieldworkers and with minimal measurement error ^8^, were converted to age and sex standardised z-scores using the WHO growth standards ^21^. Here we focus on weight-for-length data, reflective of the relative weight of a child given their stature and therefore a child’s current nutritional status ^22^, and one of the growth z-scores recommended by the WHO for diagnosing acute malnutrition ^23^. Figure 1 shows all trajectories for weight-for-length z-score (ZWfL), along with the site-specific cross-sectional means.

High within- and between-child variability is the predominant feature of the raw trajectory data, which holds from birth through 24 months and for both sites. Three trajectories are highlighted in each site, and these illustrate the dynamic complexity of each child’s growth.

### SDE models

The standard introductory text for SDEs is by Øksendal ^5^, which contains a detailed mathematical exposition of SDEs. We focus on application and SDE models are introduced through examples with technical details largely omitted. We begin with a well-studied special case SDE model which we fit to data from three individual children (separately) and compare results with a linear regression model. We then introduce a more general SDE model and fit this to data from all children from the Haydom site.

***Example 1 – Ornstein-Uhlenbeck (OU) process for a single trajectory***. Our growth outcome of interest is ZWfL, where the first observation in the growth trajectory for a single child is X _0_ when the child is aged t _0_. We now develop a model to estimate likely values of X _1_, ZWfL at a later age, t _1_. In the simplest linear context, we could formulate an expression for the mean of X _1_ conditional on the previous observation, X _0_, and the difference in age between t _1_ and t _0_. Assuming X _1_ is approximately normally distributed with mean μ and variance σ ^2^, N(μ,σ ^2^), this would give a model of the form

$${\text{X}}_{1}|{\text{X}}_{0}∼\text{N}({\text{X}}_{0}+b({\text{t}}_{1}-{\text{t}}_{0}),\sigma^{2}),\;(1)$$

which models ZWfL at a subsequent age as a linear function of the current value and the elapsed age. In this model, growth velocity – rate of change per unit time (age) – is described by *b*, a parameter to be estimated from the observed data. This is a fully specified growth model between any two time points, although too simple to be practically useful; firstly, the growth velocity, *b*, is assumed constant, and secondly, the variance σ ^2^, is constant and independent of the duration of the elapsed age between t _1_ and t _0_, whereas it might reasonably be expected that two time points closer together in age may be more similar than those further apart.

Consider the same example as above but now where we have

$${\text{X}}_{1}|{\text{X}}_{0}∼\text{N}\left({\text{e}}^{({\text{t}}_{0}-{\text{t}}_{1})\alpha }({\text{X}}_{0}-\beta )+\beta ,\frac{{\text{e}}^{-2{\text{t}}_{1}\alpha }({\text{e}}^{2{\text{t}}_{1}\alpha }-{\text{e}}^{2{\text{t}}_{0}\alpha })\sigma^{2}}{2\alpha }\right).\;(2)$$

This model now has three parameters, α, β and σ, and ( 2) is the transitional probability density function (or slice density) for the OU stochastic process. The OU model is well studied and has applications in mathematical finance ^24^ and theoretical biology ^25^. The distribution in ( 2) is a more flexible model compared to ( 1), with non-linear mean and variance terms, both of which depend on the elapsed time between t _1_ and t _0_. The OU process is typically defined in differential form by the stochastic differential equation

$${\text{dX}}_{\text{t}}=\text{α}(\text{β}-{\text{X}}_{t})\text{dt}+{\text{σdW}}_{\text{t}},\;(3)$$

where α(β − X _t_) is called the drift, σ is the diffusion and W _t_ is a Wiener (Brownian motion) process. The drift can be thought of as the slow-moving trend in growth velocity, while the diffusion is the continual perturbation of the system giving rise to volatility in velocity and therefore growth. This SDE has correlated movements through time, for example, in an OU process that commenced at time t _0_ the covariance between any two points in time, t and s, is (e ^−(s+t)α^(−e ^2t _0_α^ + e ^2α Min[s,t]^)σ ^2^)/(2α). Equation (3) provides the interpretation of the model parameters as components of the rate of change of growth, where ( 2) can be derived from ( 3) by solving the Fokker-Planck equation (see later).

**Figure 2.**
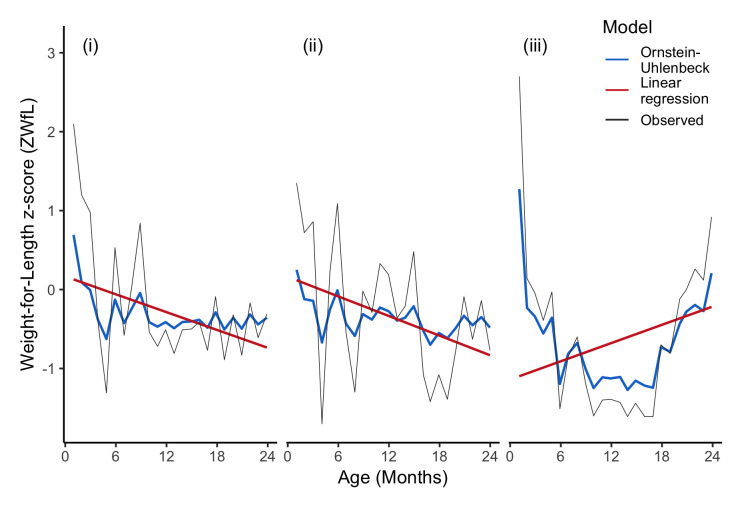
Comparison of stochastic differential equation (SDE assuming an Ornstein-Uhlenbeck process) model and linear regression model for three children from Haydom, where both models have three parameters. The difference between a curve fitting approach (fit globally) and time series approach (based on modelling the change over time) is clearly evident.

To demonstrate the practical application of an SDE model to real data, we fitted OU models to three different ZWfL trajectories from the Haydom data. We compared these with the fit of a classical linear regression (LR) model, e.g. ZWfL(t) ~ N(a _0_ + a _1_t, σ ^2^), with both models having three parameters. Figure 2 shows the raw trajectory data, along with fitted values from the OU and LR models (separately for each child). See SI.1 ( *Extended data*) ^18^ for the model fitting code. In the OU model each successive data point (say, ZWfL _1_) depends on the previous point (ZWfL _0_), which means the OU model fit is not a smooth curve, but transitions from point to point. In the LR model, the fitted value at age t _1_ does not depend directly on the previously observed value ZWfL _0_, but rather it is from a globally parameterized smooth curve computed across all observations. The OU model is a special case of a more general SDE formulation, which we present next, along with the key concepts in fitting SDEs to data.

***Defining an SDE model – basic concepts***. A general formulation for an SDE model (see 7.1.2 in Øksendal ^5^) is

$${\text{dX}}_{\text{t}}=\text{μ}(\text{t},{\text{X}}_{\text{t}},\text{θ})\text{dt}+\text{g}(\text{t},{\text{X}}_{t},\text{θ}){\text{dW}}_{\text{t}},\;(4)$$

where X _t_ is our growth outcome variable of interest (e.g. ZWfL). Functions μ(t,X _t_,θ) and g(t,X _t_,θ) generalise the drift and diffusion terms from ( 3), respectively, where θ is a set of parameters to be estimated and, as before, W _t_ is a Wiener process. The OU model from ( 3) is therefore a special case of ( 4) where μ(t,X _t_,θ)=α(β−X _t_) and g(t,X _t_,θ)=σ. Equation (4) can also be written as a stochastic integral equation

$${\text{X}}_{t}={\text{X}}_{0}+\int_{0}^{t}\mu ({\text{s},\text{X}}_{\text{s}},\theta )\text{ds}+\int_{0}^{t}\text{g}({\text{s},\text{X}}_{\text{s}},\theta ){\text{dW}}_{\text{s}},\;(5)$$

which usefully emphasises that these are models of the evolution of a continuous time stochastic process – here growth of a child. Equation (5) says that the value of growth (a random variable) for a child at *t* time units into the future from the currently observed time ( *t*=0) is the current value of growth (X _0_) plus the sum over this time interval of the drift and diffusion components of velocity. As the diffusion is integrated with respect to a “white noise” process W _s_, then X _t_ follows a probability distribution at time *t.* To make this more concrete, if ZWfL _t1_ is our growth outcome at time *t1*, then f(ZWfL _t1_) is the probability distribution of ZWfL _t1_ conditional on ZWfL _t0_, the previously observed value of growth at time *t0*. This function, f(X _t_), which is essential for model fitting, is a solution to the partial differential equation

$$\frac{\partial \text{f}}{\partial \text{t}}=\frac{\partial }{\partial \text{x}}\left(\frac{1}{2}\frac{\partial ({\text{g}}^{2}(\text{t},{\text{X}}_{\text{t}},\text{θ})\text{f})}{\partial \text{x}}-\text{μ}(\text{t},{\text{X}}_{t},\text{θ})\text{f}\right).\;(6)$$

Equation (6) is called the Fokker-Planck or forward-Kolmogorov equation (whose complete specification includes initial and boundary conditions, which have been omitted). The SDE in equation ( 4) defines the terms in Equation (6). Solving Equation (6) gives the expression for f( *X*_t_). If we consider again the OU process and plug in the relevant terms from ( 3) into ( 6) and do the necessary calculus, then we arrive at exactly the normally distributed slice density stated in ( 2).

For model fitting we need to compute the likelihood function given the slice density. If we consider first the likelihood function for trajectory data from a single child, and where we have N observations over time, then the negative log-likelihood for a single child can be written as (see Hurn ^10^)

$$-\text{log}\;ℒ(\text{θ})=-\text{log}\;\text{​}\;{\text{f}}_{0}({\text{X}}_{0}|\text{θ})-\sum_{\text{k}=0}^{\text{Ν}-1}\text{log}\;\text{f}({\text{X}}_{\text{k}+1}|{\text{X}}_{\text{k}},\text{θ}),\;(7)$$

where f _0_(X _0_|θ) is the probability density of the growth outcome variable at the first available data point, f(X _k+1_|X _k_,θ) ≡ f((X _k+1_, t _k+1_) | (X _k_, t _k_), θ) is the value of the slice density function for a stochastic process starting at (X _k_,t _k_) and evolving to (X _k+1_,t _k+1_). Equation (7) allows us to compute the likelihood function for all trajectories, including, if necessary, covariance structures across children through the inclusion of random effects (see later). One minor remark is how to deal with the first available observation, X _0_, as SDE models are defined in terms of transitions. We follow Schneider ^26^ and the existing literature in maximum likelihood estimation in SDEs and treat X _0_ as a constant, which is also typical in the time series literature.

In summary, the key steps for working with SDE models focussed on model fitting are: (i) choose a form of μ(t,X _t_,θ) and g(t,X _t_,θ) in ( 4) and then; (ii) determine the corresponding slice density which satisfies the Fokker-Planck Equation (6) and then; (iii) fit the model to the data using the likelihood function in ( 7). Here we only consider forms of μ(t,X _t_,θ) and g(t,X _t_,θ) which have known analytical solutions (slice densities) to ( 6), which then makes fitting SDE models to data no different from a standard maximum likelihood problem using standard statistical software. Mathematica software (version 11.3, Wolfram Research Inc.), for example, can be used to compute slice densities for a wide range of SDEs, and a selection of these solutions is provided for reference in SI.2 (see *Extended* data) ^18^ as illustration. While in theory it is possible to fit SDEs that do not have an analytical solution to (6) to data, in practice this is numerically challenging (see the Discussion).

***Example 2 - OU process for multiple trajectories***. In the Haydom data we have 224 trajectories (children) across 0–24 months, and we now fit (non-linear) mixed model variants of the OU process, along with standard linear mixed models to these data. Table 1 gives a summary of different parameterizations and goodness of fit metrics. The modelling code is provided in SI.3a along with model output SI.3b (see *Extended data*) ^18^.

**Table 1.** Model goodness of fit comparisons using a selection of stochastic differential equation and linear regression models.

Model	No. parameters	Remarks	AIC (smaller is better)	BIC (smaller is better)
Ornstein-Uhlenbeck ( Equation 3)	3	No random effects	**12181**	**12201**
Linear regression			15454	15473
Ornstein-Uhlenbeck with mixed effects	6	Random speed of reversion ^1^ and long-term mean with covariance ^2^	**11544**	**11564**
Linear mixed effects regression		Random intercept and slope with covariance ^2^	11607	11627

AIC, Akaike information criterion; BIC, Bayesian information criterion. . ^1^α in the Wiener (Brownian motion) process and is the covariance between time points (see text above for detail); ^2^Child-level random effects.

For the same number of parameters, the OU process gives substantially better Akaike information criterion (AIC) and Bayesian information criterion (BIC) metrics, and fitting these SDE models including random effects is straightforward, requiring only with a few lines of code in SAS's proc nlmixed. These mixed models can also be implemented in the Stan ^27^ language, with an OU specific example using Stan provided by Goodman (2018) ^28^.

### Main case study - model formulations

Our main results comprise of an illustrative case study where we considered the combined data from Haydom and Venda. The general model formulations considered, and model search process are detailed below. To keep the analysis as clear as possible we considered only one covariate, (in addition to age) in the modelling, a categorical variable indicating site.

***Linear mixed models (LMMs)***. We considered LMMs where the most general formulation for ZWfL (response) was: a polynomial function of age (continuous) and site (two categories); with interactions between age and site; fixed and random (normally distributed) effects for the age terms in the polynomial; where random effects for the age terms (including intercept) had an unstructured covariance matrix; and within child errors had an AR(1) – autoregressive first order – covariance structure to allow serial dependence between errors. Increasing orders of polynomial (up to fourth order) were examined, guided by AIC and BIC metrics, in addition to trimming terms with high p-values (>0.1). These model formulations can be readily fitted to trajectory data using proc mixed in SAS. Relevant SAS code is provided in SI.4a (see *Extended data*) ^18^. This can also be achieved using Stan, for example adapting Goodman (2018) ^28^.

***SDE (non-linear) mixed models***. The most general formulation considered for the SDE models was

$$\text{d}{\text{X}}_{t}=({\text{α}}_{2}+{\text{α}}_{3}\text{t}+{\text{α}}_{4}{\text{t}}^{2}+{\text{α}}_{5}t^{3}+{\text{α}}_{1}X_{t})\text{dt}+\text{σ}dW_{t},\;(8)$$

where X _t_ is ZWfL at age *t* and so the rate-of-change per unit time for ZWfL depends on both the current age of the child and the child’s current value of ZWfL. Specifically, we considered a polynomial (a cubic in equation 8) function of age, plus a linear term (α _1_X _t_) in ZWfL. This polynomial formulation of SDE in ( 8) has a closed form of slice density (a normal distribution) if we hold (α _1_, α _2_, α _3_, α _4_, α _5_, σ) constant. Mathematica was used to determine the slice density which is

$$\begin{array}{l} {\text{X}}_{1}|{\text{X}}_{0}∼\text{N}(\frac{1}{\alpha_{1}^{4}}e^{-t_{0}\alpha_{1}}(e^{t_{1}\alpha_{1}}x_{0}\alpha_{1}^{4}-6(e^{t_{0}\alpha_{1}}-e^{t_{1}\alpha_{1}})\alpha_{5}+\alpha_{1}(-2(e^{t_{0}\alpha_{1}}-e^{t_{1}\alpha_{1}})\alpha_{4}-6(-e^{t_{1}\alpha_{1}}t_{0}\\ \;+e^{t_{0}\alpha_{1}}t_{1})\alpha_{5})\;+\;\alpha_{1}^{2}((-e^{t_{0}\alpha_{1}}+e^{t_{1}\alpha_{1}})\alpha_{3}+\;2e^{t_{1}\alpha_{1}}t_{0}\alpha_{4}+3e^{t_{1}\alpha_{1}}t_{0}^{2}\alpha_{5}\\ \;-e^{t_{0}\alpha_{1}}t_{1}(2\alpha_{4}+3t_{1}\alpha_{5}))+\alpha_{1}^{3}((-e^{t_{0}\alpha_{1}}+e^{t_{1}\alpha_{1}})\alpha_{2}+e^{t_{1}\alpha_{1}}t_{0}\alpha_{3}+e^{t_{1}\alpha_{1}}t_{0}^{2}\alpha_{4}\\ \;+e^{t_{1}\alpha_{1}}t_{0}^{3}\alpha_{5}-e^{t_{0}\alpha_{1}}t_{1}(\alpha_{3}+t_{1}(\alpha_{4}+t_{1}\alpha_{5})))),\frac{(-1+e^{2(-t_{0}+t_{1})\alpha_{1}})\sigma^{2}}{2\alpha_{1}}). \end{array}\;(9)$$

Comparing Equation (8) with ( 9) demonstrates how compact the differential form of the model is, but it is the slice density which is required for model fitting and parameter estimation. The most general form of ( 8) we considered additionally allowed each of (α _1_, α _2_, α _3_, α _4_, α _5_, σ) to be included in the model as both a fixed and random (normally distributed) effect, thereby allowing trajectories to be tailored to individual children. An unstructured covariance matrix for the random effects with simplifications down to a diagonal covariance matrix were considered. The model search considered increasing orders of polynomial up to cubic and in keeping with the LMM search was guided by AIC and BIC metrics, in addition to trimming terms with high p-values (>0.1). Relevant SAS code is provided in SI.4a (see *Extended data*) ^18^. This can also be achieved using Stan, for example, adapting Goodman (2018) ^28^.

### Prediction

The main real-world application area of SDEs is in predictive modelling (e.g. Iversen *et al.* ^26^). Here we use SDE models to predict future growth given a child’s current age and current ZWfL. Such predictions have two application areas: 1) as part of an individual child’s risk assessment to determine if they require an intervention; and 2) to elucidate structure hidden within the large variability across growth trajectories, which may then offer new insights into drivers of growth at the population level. We use our best fitting SDE model to predict future growth trajectories across a grid of starting points for age and ZWfL, separately for each of the two sites.

Predictions are calculated using a 10-fold random sampling approach, where we draw from all the parameters estimated (fixed and random) in the best fitting SDE model – one set for each child. Which parameter sets are chosen to generate predictions depends on how likely trajectories generated from each set are to have visited each given starting point across an age-ZWfL grid. This adds an important element of “locality” to our predictions, combined with 10-fold sampling to provide an indication of robustness of our predictions. A more detailed description of the prediction algorithm is given below, with full R code provided in SI.5 (see *Extended data*) ^18^.

For each point across an age-ZWfL grid we compute the likelihood of observing this point for each set of parameters, using the relevant slice density, where the initial starting point is the first age available for each trajectory. Predictions from each grid point progress through increasing ages using the new slice distribution at each next point in time. For example, for predictions in Venda we have n=236 likelihood values for each age-ZWfL grid point. The most likely parameter set is then used for the prediction of the next ZWfL at the next age, with 10-fold sampling used to indicate how robust this prediction is. The 10-fold sampling splits these n=236 parameter sets into 10 random groups, and within each group we choose the parameter set with the highest likelihood value as the one to be used for the next prediction. In summary, from a fixed starting point in an age-ZWfL grid we have a main prediction at each future age up to 24 months, plus 10 additional predictions at each age as a sensitivity analysis (incrementing age in small steps).

## Results

### Linear mixed models and SDE mixed models

Using individual child trajectory data over 0–24 months for ZWfL from all n=460 children from the two sites, the best fitting LMM model was a cubic polynomial with a single interaction term between age squared and site (with no separate term for site), which gave AIC=21913 and BIC=21983. The best fitting SDE model had corresponding values of AIC=21718 and BIC=21772, where this model had random effects in four of the six model parameters, a diagonal covariance matrix and site included in two of the drift parameters and the diffusion parameter. Full modelling descriptions can be found in SI.4a and results, including parameter estimates, can be found in the SI4.b (see *Extended data*) ^18^. The AIC and BIC metrics suggest the SDE offers an improved fit to the data. Examining residuals and fitted values the fit of each model is visually similar; however, there are notable qualitative and quantitative differences.

**Figure 3.**
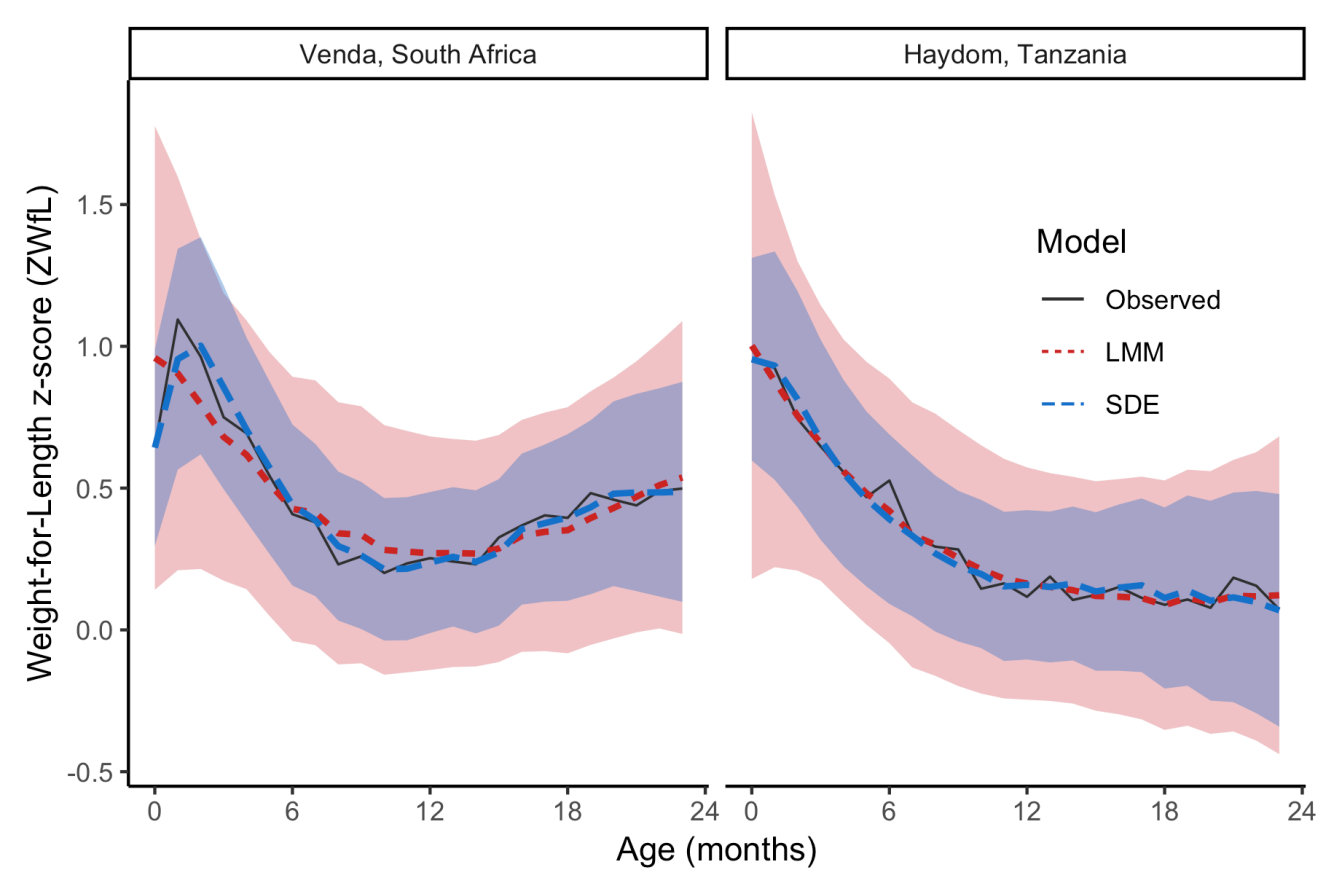
Comparison between population means estimated from stochastic differential equation (SDE) and linear mixed models (LMM) with 95% confidence intervals, and observed monthly means. SDE model has narrower confidence intervals in each site, entirely contained within the wider LMM confidence interval.

Figure 3 shows observed monthly means compared with estimates of the population mean ZWfL from each model. These are quantitatively similar except that the SDE correctly captures the initial shape of the mean, a short rapid increase then decrease during the first six months in the Venda data, whereas the LMM estimates a steady decrease from birth through six months. Figure 3 also shows confidence intervals for the population mean of ZWfL from each model over age; the SDE model has considerably narrower intervals suggesting it has explained more of the variation in the data than the LMM model.

In summary, our results so far suggest that our SDE model is at least as good, and appears superior in some respects, to a reasonable choice of classical LMM.

### Prediction

**Figure 4.**
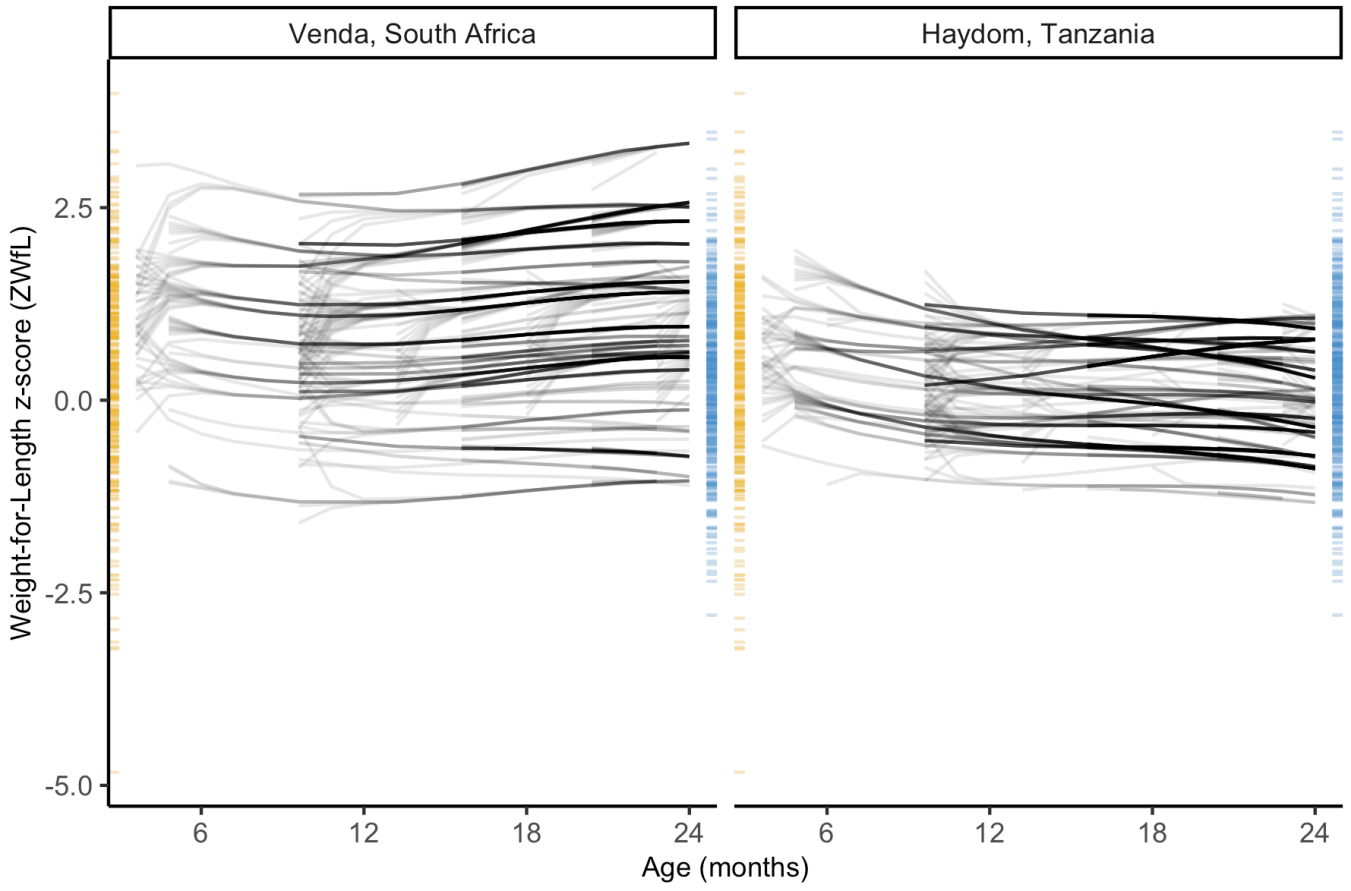
Predicted future weight-for-length trajectories from a grid of starting points using an SDE model. Yellow ticks indicate the observed weight-for-length at 0 months; blue ticks indicate the observed weight-for-length at 24 months. The best-fitting predictions are shown in red and 10-fold cross validation in grey. The distributions of observed weight-for-length at both zero and 24 months in each site is shown to the right of the respective plot. The predictions appear to cluster together into a smaller set of “paths”, which also differ between sites.

Figure 4 shows predictions of the most likely future ZWfL trajectory for a child, conditional on current age and ZWfL values. A grid of starting values for age and ZWfL within the ranges observed (from both sites) was used, with separate predictions for each site. There are several particularly striking features in these predictions; most notably, they suggest the presence of longitudinal clustering within each site, as we find what appear to be a small distinct set of “paths” or “streams” hidden inside the large between-child variability in trajectories observed in Figure 1. These clusters also appear to differ quantitatively and qualitatively between sites, with fewer clusters in Haydom and of different shapes to those in Venda. Further, these clusters imply some degree of canalisation of trajectories, for example, the small number of children who are wasted with ZWfL values of ≤ -2 (Venda, n = 16/236; Haydom, n = 2/224) converge on similarly low predicted values (the rapid increase from extreme values [e.g. ≤ -3] shows that predictions return to values where more data are present).

## Discussion

We have presented a novel approach for analysing child growth trajectories, using a modelling methodology, stochastic differential equations, widely used in other fields but not yet in child growth research. The use of a continuous time stochastic process approach, such as SDEs, to model child growth trajectories is conceptually appealing as it explicitly acknowledges - through drift and diffusion processes - the highly complicated dynamic environment into which a new born child is delivered and exposed, particularly in resource-limited settings. Our results show that SDEs also have practical appeal as they offer very different (highly non-linear) formulations from the usual additive linear models, which gave good results with our case study data. This suggests that SDEs may be an attractive alternative to other established methods, at least as supporting analyses, moreover because SDEs can also be readily fitted using standard software such as SAS or open source alternatives such as Stan. Our supplementary information contains modelling code that can adapted to other study data ^18^ or as a basis for comparison to other methods and alternative drift/diffusion terms.

While only an initial exploration of a subset of the MAL-ED data using SDE modelling, our predictive results presented in Figure 4 were both unexpected and exciting. These results suggest that the predictive capability of SDE models could potentially reveal new insights hidden by the large between-child variations that typify child growth. For example, here the prediction of ZWfL implies canalisation of growth trajectories, with particular implications for children who start life wasted (or close to) and are predicted to remain so through the first two years of life, although it is worth noting that observations of wasting were rare in these two populations. The predictive method presented is relatively ad-hoc and simple – prediction in SDEs with random effects is novel - and is an area in need of development.

We restricted our presentation to a narrow selection of simple SDE models, many more parameterizations are available with explicit expressions for the slice density (e.g. using software like Mathematica). More complex formulations, particularly for the diffusion function g(t,X _t_,θ), may add considerable richness to an SDE model’s dynamic behaviour, for example, by incorporating additional covariates such as seasonality or food security, morbidity or even measurement error; however, these would require numerical methods to compute the likelihood function. Initial explorations suggest this is far from straightforward, both in terms of computational feasibility and also numerical stability, and is another area ripe for future development.

## Data availability

### Underlying data

Data from the MAL-ED study are available from https://clinepidb.org/. Guest users can view data and access analysis tools and record pages, but must obtain approval from the data providers to download data. The request may be submitted via a form that pops up when a user logs in with a registered account and clicks “Download data”.

### Extended data

Zenodo: Introducing a drift and diffusion framework for childhood growth research. https://doi.org/10.5281/zenodo.4278002 ^18^.

This project contains the following extended data:

- SI1.pdf (Model code for OU and linear regression models, in SAS)
- SI2.pdf (Illustrative reference slide densities, using Mathematica)
- SI3a.pdf (SAS code to compare OU and LMM models for Table 1)
- SI3b.pdf (SAS model output comparing OU and LMM models in Table 1)
- SI4a.pdf (SAS code to fit mixed effects OU and LMM models)
- SI4b.pdf (SAS model output for OU and LMM models)
- SI5.pdf (R code to generate figures, including the prediction algorithm to generate Figure 4)

Data are available under the terms of the Creative Commons Attribution 4.0 International license (CC-BY 4.0).

### Code availability

Source code available from: https://github.com/fraseriainlewis/gatesopenresearch_SI

Archived code at time of publication: https://doi.org/10.5281/zenodo.4278002 ^18^

License: Creative Commons Attribution 4.0 International license (CC-BY 4.0).
