# Supplementary material for: Introducing a drift and diffusion framework for childhood growth research
Source: Gates Open Res. 2020 Nov 26;4:71. Originally published 2020 Jun 29. [Version 2] doi: 10.12688/gatesopenres.13123.2 (PMC7791186; doi:10.12688/gatesopenres.13123.2)
Supplement: Peer review history

# Peer review: Reviewer response for version 2

**DOI:** 10.21956/gatesopenres.14421.r30075

**Published:** 2021-01-07

**Authors:** Noel Cameron

**Affiliations:** 1 School of Sport, Exercise and Health Sciences, Loughborough University, Loughborough, UK

I think that the submission can go forward because it is scientifically valid although I still am not convinced that this approach is useful or that it makes a significant contribution to what is already known. I don’t think this modelling does provide greater insight into anthropometric data and don’t think that authors have made a case for its significance over other methods. I remain to be convinced that it will be widely used in the analysis of growth data.

Is the work clearly and accurately presented and does it cite the current literature?

Partly

If applicable, is the statistical analysis and its interpretation appropriate?

I cannot comment. A qualified statistician is required.

Are all the source data underlying the results available to ensure full reproducibility?

Yes

Is the study design appropriate and is the work technically sound?

Yes

Are the conclusions drawn adequately supported by the results?

Partly

Are sufficient details of methods and analysis provided to allow replication by others?

Yes

Reviewer Expertise:

NA

I confirm that I have read this submission and believe that I have an appropriate level of expertise to confirm that it is of an acceptable scientific standard.

# Peer review: Reviewer response for version 2

**DOI:** 10.21956/gatesopenres.14421.r30076

**Published:** 2020-12-03

**Authors:** James A. Berkley

**Affiliations:** 1 The Childhood Acute Illness and Nutrition (CHAIN) Network, Nairobi, Kenya; 2 University of Oxford, Oxford, UK; 3 KEMRI/Wellcome Trust Research Programme, Kilifi, Kenya

Thank you, my comments have been well-addressed and I believe the manuscript is suitable for indexing.

Is the work clearly and accurately presented and does it cite the current literature?

Yes

If applicable, is the statistical analysis and its interpretation appropriate?

I cannot comment. A qualified statistician is required.

Are all the source data underlying the results available to ensure full reproducibility?

Yes

Is the study design appropriate and is the work technically sound?

Yes

Are the conclusions drawn adequately supported by the results?

Partly

Are sufficient details of methods and analysis provided to allow replication by others?

Yes

Reviewer Expertise:

Child survival, infectious diseases, malnutrition, newborn health, clinical trials, anthropometry

I confirm that I have read this submission and believe that I have an appropriate level of expertise to confirm that it is of an acceptable scientific standard.

# Peer review: Reviewer response for version 1

**DOI:** 10.21956/gatesopenres.14305.r29206

**Published:** 2020-08-10

**Authors:** James A. Berkley

**Affiliations:** 1 The Childhood Acute Illness and Nutrition (CHAIN) Network, Nairobi, Kenya; 2 University of Oxford, Oxford, UK; 3 KEMRI/Wellcome Trust Research Programme, Kilifi, Kenya

This is an interesting and novel methods paper outlining an approach to analyzing child growth through translation of the SDE approach from physics and financial modelling. SDEs extend the concept common to growth modelling methods that individuals vary stochastically about a population curve.

  

- It would be helpful in the introduction to outline which uses the SDE approach is aiming at. For example, descriptive, explanatory or forecasting in populations or real-time use in individuals.
- In the introduction, for clarity please also include whether ‘drift’ is also thought to represent exposures such as seasonal food security and dietary intake patterns and feeding practices that may change with age (e.g. breastfeeding) which may be more influential than the described ‘developmental biological aspects’.
- Reference 8 suggests that good reliability weight and length can be assumed, however length is more difficult to measure precisely in younger infants and I notice extreme values appear more common close to the time of birth on the figures, including 4 of the 6 individuals presented in figures 1 and 2. Translation to Z scores (a ratio) may compound errors. Was reliability assessed for (computer calculated) ZWfL? If the data are available, I suggest including it in this report, including performance across the age range 0-24m. It would also be worth commenting that apparent weight-for-length may be affected by hydration status as both may provide additional stochastic variability that varies with age. The former may potentially affect the constant term X _0_ and the starting points across the age-ZWfL grid.
- An issue worth considering is that when examining changes in Z scores is that the standard deviation (the denominator for ZWfL) varies across the range of length (see: https://www.who.int/childgrowth/standards/wfl_girls_0_2_zscores.txt). This means that children with a constant deficit in weight-for-length as they growth longer/heavier may demonstrate changes in ZWfL. Leroy *et al. *(2015 ^1^) proposed the use of an absolute difference criterion (for HAZ in their case) as a more robust way to assess changes in nutritional status.
- The formulations of the SDE models themselves is outside my expertise.
- In Methods, example 2, it may be helpful to the readers to point out if child ID was a random effect term in the non-linear mixed model variants and explain ‘random speed of reversion’.
- The longitudinal clustering is very interesting, with potential genetic and environmental interpretations. However, the finding would be strengthened by validation using another method such as simple probability estimates for ending up in one of the final ‘bins’ identified in the model to reassure readers this is not an artifact of the method (quantization?) or due to different error in the first measurements (as above).
- The discussion should expand on future research that the method leads to. Anthropometry is a practical proxy for health and nutritional status and validation of criteria for intervention are typically based on mortality risk in large populations. As a next stage of research, prediction of actual health events such as death, serious illness or impaired neurodevelopment would be valuable to mention. Additionally, it would be helpful to comment on the models’ ability to include time-varying covariates such as seasonality, illness or food security shock, as well as preterm birth.
- The narrower CIs suggest the SDE model is a better approximation of the data than the linear mixed models. However, linear models may not be regarded as a current gold standard for growth models, and comparisons with others including latent growth models, SITAR, multilevel fractional polynomial models or penalised splines etc. This is a limitation of the paper and area for future work.

Is the work clearly and accurately presented and does it cite the current literature?

Yes

If applicable, is the statistical analysis and its interpretation appropriate?

I cannot comment. A qualified statistician is required.

Are all the source data underlying the results available to ensure full reproducibility?

Yes

Is the study design appropriate and is the work technically sound?

Yes

Are the conclusions drawn adequately supported by the results?

Partly

Are sufficient details of methods and analysis provided to allow replication by others?

Yes

Reviewer Expertise:

Child survival, infectious diseases, malnutrition, newborn health, clinical trials, anthropometry

I confirm that I have read this submission and believe that I have an appropriate level of expertise to confirm that it is of an acceptable scientific standard, however I have significant reservations, as outlined above.

## References

[1] : Using height-for-age differences (HAD) instead of height-for-age z-scores (HAZ) for the meaningful measurement of population-level catch-up in linear growth in children less than 5 years of age. *BMC Pediatr*.2015;15: 10.1186/s12887-015-0458-9 145 10.1186/s12887-015-0458-9 26444012PMC4595313

# Response

**Published:** 2020-11-17

**Authors:** Ben McCormick

**It would be helpful in the introduction to outline which uses the SDE approach is aiming at. For example, descriptive, explanatory or forecasting in populations or real-time use in individuals.**

 We have amended the introduction to note that we believe this approach to be particularly useful in explanatory analysis of growth data (“SDEs can enhance mechanistic interpretation of the drivers of variability”). We note that in the discussion we do discuss the advantage SDEs offer to forecasting because they are process-based models rather than purely descriptive. Forecasts ought, assuming appropriate covariables, better account for variance.

**In the introduction, for clarity please also include whether ‘drift’ is also thought to represent exposures such as seasonal food security and dietary intake patterns and feeding practices that may change with age (e.g. breastfeeding) which may be more influential than the described ‘developmental biological aspects’.**

 We have added these to the text, but we note that the terms that are included in the drift and diffusion are specific to the conceptual model examined. Certainly, these terms could be included (in either or both components) and it is a matter of the appropriate technical details. This reflects the richness of the method, because such exposures can be explored mechanistically. Diet might conceivably impact both drift (‘usual’ diet) or diffusion (seasonal food insecurity).

**Reference 8 suggests that good reliability weight and length can be assumed, however length is more difficult to measure precisely in younger infants and I notice extreme values appear more common close to the time of birth on the figures, including 4 of the 6 individuals presented in figures 1 and 2. Translation to Z scores (a ratio) may compound errors. Was reliability assessed for (computer calculated) ZWfL? If the data are available, I suggest including it in this report, including performance across the age range 0-24m. It would also be worth commenting that apparent weight-for-length may be affected by hydration status as both may provide additional stochastic variability that varies with age. The former may potentially affect the constant term X _0_ and the starting points across the age-ZWfL grid.**

 Reliability (noted in reference 8) was measured on the basis of 5% remeasurement. Reliability was indeed lower for measurements of younger children, and lower for the combined WFL than either weight or length, albeit with correlation coefficients over a range of 0.92 (1 month) to 0.98 (24 months). We have noted that this could be incorporated into the model.  

**An issue worth considering is that when examining changes in Z scores is that the standard deviation (the denominator for ZWfL) varies across the range of length (see: https://www.who.int/childgrowth/standards/wfl_girls_0_2_zscores.txt). This means that children with a constant deficit in weight-for-length as they growth longer/heavier may demonstrate changes in ZWfL. Leroy *et al. *(2015 ^1^) proposed the use of an absolute difference criterion (for HAZ in their case) as a more robust way to assess changes in nutritional status.**

 The Leroy et al. argument is particularly interesting in the context of recovery from stunting, which tends to occur post-24 months. For this particular study, WFL was used as an example and the methodology would be equally applicable to other anthropometric metrics.

**The formulations of the SDE models themselves is outside my expertise.** 

 It is our hope that this introduction may inspire others to consider this approach and that it will make the methods less opaque by demonstrating their application and accessibility in conventional software.

**In Methods, example 2, it may be helpful to the readers to point out if child ID was a random effect term in the non-linear mixed model variants and explain ‘random speed of reversion’.**

 We have now noted that these were child-level parameters (random effects). The random speed of reversion is the covariance between time points, so the closer time points are the more similar they are. We have added a note to the text.

**The longitudinal clustering is very interesting, with potential genetic and environmental interpretations. However, the finding would be strengthened by validation using another method such as simple probability estimates for ending up in one of the final ‘bins’ identified in the model to reassure readers this is not an artifact of the method (quantization?) or due to different error in the first measurements (as above).**

 We apologise that we mis-wrote the code for the prediction presented in figure 4. The model to evaluate predictions was correct, but the 10-fold selection of the best fitting points incorrectly selected observations, not trajectories. We have updated the figure, which more accurately captures the density of observations and have added a ‘rug plot’ to the figure, with marks to indicate the observed WFL at months zero and 24.

 It is still the case the trajectories tend to cluster into a smaller number of ‘streams’ as different initial conditions tend to converge, but to a much lesser extent.

**The discussion should expand on future research that the method leads to. Anthropometry is a practical proxy for health and nutritional status and validation of criteria for intervention are typically based on mortality risk in large populations. As a next stage of research, prediction of actual health events such as death, serious illness or impaired neurodevelopment would be valuable to mention. Additionally, it would be helpful to comment on the models’ ability to include time-varying covariates such as seasonality, illness or food security shock, as well as preterm birth.** 

 This is an interesting idea and perhaps the opposite way to how we were framing the question (looking at how events during childhood manifest in growth outcomes rather than growth as a predictor of later outcomes). Individual-level coefficients from the model could indeed be used as parameters to predict other health outcomes. We have noted the incorporation of seasonal parameters.

**The narrower CIs suggest the SDE model is a better approximation of the data than the linear mixed models. However, linear models may not be regarded as a current gold standard for growth models, and comparisons with others including latent growth models, SITAR, multilevel fractional polynomial models or penalised splines etc. This is a limitation of the paper and area for future work.**

 It is not clear that there is a gold standard (linear mixed models are still widely used) as such and as Reviewer 1 notes, there is not much to pick between such models that are fundamentally similar in concept (e.g. fitting to the mean using some variant of least squares). We note that the accessibility of the method in conventional software might mean that this approach can be run in parallel for routine comparisons. That said, we have noted this important future avenue in the discussion.

# Peer review: Reviewer response for version 1

**DOI:** 10.21956/gatesopenres.14305.r28999

**Published:** 2020-08-04

**Authors:** Noel Cameron

**Affiliations:** 1 School of Sport, Exercise and Health Sciences, Loughborough University, Loughborough, UK

1. The authors need to explain why yet another model for growth is required or necessary or advantageous. 
2. Introduction: “One of the main barriers to analysing child growth data is that individual growth trajectories display highly variable and complicated dynamic behaviour, differing markedly between children, even from the same geographic and socio-economic group. As such, developing growth models from which actionable insights can be extracted – such as identification of interventions at the population level or predictive risk assessments at individual child level – is both methodologically and practically challenging.”
  The variability demonstrated between children is not a “barrier” to analysing growth data.  The variability is a measure of the variation in normal growth rates and in response to potential constraints on growth. To smooth out or diminish this variation without investigation destroys the very essence of growth studies particularly in clinical scenarios.   
3. “WHO reference standards” – there is no such thing as a “reference standard”. Growth charts are either based on cross-sectional large sample data and are “references” or selected longitudinal samples and are “standards”. The WHO charts are standards.
4. Whilst I am impressed by the comparison between the OU method and LMM in Figure 4, the lack of any apparently significant improvements or insights into the growth trajectory makes me wonder if the new method really does represent a significant advantage over previous methods.
5. I would like the authors to explore what precisely is gained by applying this method as opposed to previous methods. Does it allow earlier detection of poor growth? Does it provide a more reliable prediction of future growth? If used as an intervention trigger what preceding data are required and prediction accuracy is obtained?
6. References include only minimal publications relating to growth modelling. 

Is the work clearly and accurately presented and does it cite the current literature?

Partly

If applicable, is the statistical analysis and its interpretation appropriate?

I cannot comment. A qualified statistician is required.

Are all the source data underlying the results available to ensure full reproducibility?

Yes

Is the study design appropriate and is the work technically sound?

Yes

Are the conclusions drawn adequately supported by the results?

Partly

Are sufficient details of methods and analysis provided to allow replication by others?

Yes

Reviewer Expertise:

Human growth and development - Auxology

I confirm that I have read this submission and believe that I have an appropriate level of expertise to confirm that it is of an acceptable scientific standard, however I have significant reservations, as outlined above.

# Response

**Published:** 2020-11-17

**Authors:** Ben McCormick

**The authors need to explain why yet another model for growth is required or necessary or advantageous.**

 This is an important consideration true of any modelling work. Given that models can serve a multitude of purposes in this case, we believe that this alternative modelling strategy (rather than the model results *per se*) is an opportunity to gain greater insight from anthropometric data. Growth models of the same data will (should!) arrive at very similar fitted values, however, the SDE approach is conceptually different (and has correspondingly different math). In this case, the SDE partitions variance between two different mechanistic phenomena and therefore offers an interpretation that is not available to more traditional curve-fitting approaches that describe the pattern, but perhaps not the underlying process. We have added text to the introduction to this effect.

**The variability demonstrated between children is not a “barrier” to analysing growth data.  The variability is a measure of the variation in normal growth rates and in response to potential constraints on growth. To smooth out or diminish this variation without investigation destroys the very essence of growth studies particularly in clinical scenarios.  ** 

 We have revised the text to “challenges” and thank the reviewer for their comment. The principle purpose of an SDE model is that it explicitly describes variability in growth rates rather than fitting a population mean and treating variance as a nuisance parameter. SDEs are most often used where a process has noise and variability, as the reviewer notes, is the case with growth modelling. In this sense, the SDE is the perfect match to produce better (more robustly capturing uncertainty) forecasts of growth. We have amended the text to better draw out this point (“more accurately capturing external sources of variability and uncertainty”).

**“WHO reference standards” – there is no such thing as a “reference standard”. Growth charts are either based on cross-sectional large sample data and are “references” or selected longitudinal samples and are “standards”. The WHO charts are standards.**

 Thank you for this clarification, we have amended the text accordingly.

**Whilst I am impressed by the comparison between the OU method and LMM in Figure 3, the lack of any apparently significant improvements or insights into the growth trajectory makes me wonder if the new method really does represent a significant advantage over previous methods.**

  We illustrate an improved model fit using a relatively simple model akin to Brownian motion. This manuscript lays out an introduction to the approach, but the SDE method is much richer in terms of capturing a dynamic stochastic process.

**I would like the authors to explore what precisely is gained by applying this method as opposed to previous methods. Does it allow earlier detection of poor growth? Does it provide a more reliable prediction of future growth? If used as an intervention trigger what preceding data are required and prediction accuracy is obtained?**

 As with the first question posed by reviewer 2, we have noted that the principle advantage of the SDE approach is the descriptive partitioning of variance (“SDEs can enhance mechanistic interpretation of the drivers of variability (both long- and short-term) in growth”). In that sense, we believe this method can (with appropriate terms) offer superior mechanistic insight: variables can relate to one or both of the drift and shift components. With such insight, one might more appropriately distinguish between factors relating to growth trends and those relating to short-term fluctuations (albeit these fluctuations can have lasting consequences if negative effects aren’t removed).

**References include only minimal publications relating to growth modelling.**

 Most published growth models take a very similar methodological approach (curve-fitting) whereas out intention was to introduce a conceptually different approach rather for contrast.
